# Whooping Cough Treated without Drugs

**Published:** 1900-09

**Authors:** 


### Whooping Cough Treated Without Drugs.

Norton (Arch. of Ped.) reports a series of cases treated by the rectal administration of carbonic acid gas, as first used by Bergeon. In a wide-mouthed bottle, holding a pint or more, and supplied with a perforated cork through which a glass tube extends, is placed six drachms of bicarbonate of sodium. The bottle is then filled about one-third full of water and about one-half ounce of crystals of tartaric acid added. The gas is conducted to the rectum by a rubber tube and a suitable nozzle. The treatment was given three times daily, and lasted five to ten minutes at each seance. It is explained that the extra amount of carbonic acid added in this way to the blood requires an extra amount of oxygen to reach the air vesicles. One hundred and fifty children had this treatment; 143 were apparently benefited. The vomiting ceased by the second or third day. The number of paroxysms was reduced and the severity diminished.

Dr. Norton calls attention to the use of the laryngeal tube in whooping cough as suggested by Dr. O'Dyer shortly before his death. This he considered a justifiable procedure in grave cases of the disease. In using the "retained tube" the hard rubber instrument should be used, since calcareous matter may form in a metal tube and do serious injury to the mucous membrane. Three cases have come under Dr. Norton's observation. The effect was interesting. There would appear the signs of an impending paroxysm, excitement and the desire to hold on to something for support. Then followed a spasmodic cough of the usual length as far as expiration was concerned, sometimes even causing cyanosis. But when the inspiratory effort was made there could be no glottic spasm, the air entered the lungs freely through the tube, and the paroxysm terminated abruptly without the least distress or vomiting.—*Medical Sentinel,* Portland, Oregon.

